# Stealth invaders: unraveling the mystery of neurotropic viruses and their elusive presence in cerebrospinal fluid – a comprehensive review

**DOI:** 10.1097/MS9.0000000000000736

**Published:** 2023-04-27

**Authors:** Bahadar S. Srichawla, Vincent Kipkorir, Muhammad Romail Manan, Arkadeep Dhali, Sebastian Diebel, Tirtha Sawant, Subtain Zia, Diego Carrion-Alvarez, Richard C. Suteja, Khulud Nurani, Mihnea-Alexandru Găman

**Affiliations:** a Department of Neurology; b Department of Infectious Diseases, University of Massachusetts, Chan Medical School, Massachusetts, USA; c Services Institute of Medical Sciences, Shadman, Lahore, Punjab, Pakistan; d Department of Internal Medicine, Nottingham University Hospitals NHS Trust, Nottingham, United Kingdom; e Department of Family Medicine, Northern Ontario School of Medicine University, Sudbury, Canada; f Department of Neurology, Spartan Health Sciences University, Spartan Drive St. Jude’s Highway, St. Lucia; g Department of Human Anatomy and Physiology, University of Nairobi, University Way, Nairobi, Kenya; h Faculty of Medicine, Udayana University, Kampus BukitKabupaten Badung, Bali, Indonesia; i Departmento de Medicina Interna, ISSSTE Regional de Monterrey, Monterrey, Nuevo Leon, Mexico; j Faculty of Medicine, “Carol Davila” University of Medicine and Pharmacy, Bucuresti, Romania; k Romania and Department of Hematology, Center of Hematology and Bone Marrow Transplantation, Fundeni Clinical Institute, Soseaua Fundeni 258, Bucuresti, Romania

**Keywords:** arboviruses, flaviviruses, neuroinvasion, neurotropism, transneuronal spreadtrojan horse, west nile virus, zika virus, zoonoses, zoonosis

## Abstract

Neurotropic viruses are a threat to human populations due to ongoing zoonosis. A wide array of neurological manifestations can occur most often including parkinsonism, encephalitis/encephalopathy, flaccid myelitis, and Guillain-Barré syndrome. Neuroinvasion occurs through: transneural transmission, blood brain barrier (BBB) dysfunction, and ‘trojan horse’ mechanism or infected immune cell trafficking into the central nervous system (CNS). Transneural transmission occurs through virus mediated hijacking of intracellular transport proteins allowing retrograde viral transport. BBB dysfunction occurs through cytokine storm increasing membrane permissibility. Increased chemokine expression allows leukocyte trafficking to the BBB. Virally infected leukocytes may successfully pass through the BBB allowing the pathogen to infect microglia and other CNS cell types. We define cerebrospinal fluid (CSF) nondetection as a virus’ ability to evade direct CSF detection but still causing significant neurological symptoms and disease. Mechanisms of CSF nondetection include: transneuronal propagation through trans-synaptic transmission, and synaptic microfusion, as well as intrathecal antibody synthesis and virus neutralization. Direct virus detection in CSF is associated with an increased neurological disease burden. However, the lack of CSF detection does not exclude CNS involvement due to possible neuroevasive mechanisms.

## Introduction

Highlights

- Transneural transmission occurs through virus mediated hijacking of intracellular transport proteins allowing retrograde viral transport.
- Blood brain barrier dysfunction occurs through cytokine storm increasing membrane permissibility.
- Cerebrospinal fluid (CSF) nondetection is a virus’ ability to evade direct cerebrospinal fluid detection but still causing significant neurological symptoms and disease.
- Mechanisms of CSF nondetection include: transneuronal propagation through trans-synaptic transmission, and synaptic microfusion, as well as intrathecal antibody synthesis and virus neutralization.
- Direct virus detection in CSF is associated with an increased neurological disease burden. However, the lack of CSF detection does not exclude central nervous system involvement due to possible neuroevasive mechanisms.

During the past 50 years, numerous viral epidemics have emerged around the world. This includes the West Nile virus (WNV), the dengue virus, and most recently the Zika virus throughout the Americas^1,2^. Systemic symptoms such as fever, myalgias, and arthralgias are commonly reported. However, neurotropism or neuroinvasion is of significant concern for neuroinfectious disease specialists due to their varying presentation, morbidity, mortality, and lack of effective treatment options^3^. For example, prior strains of the Zika virus did not exhibit neurotropic effects. However, starting in 2016, neurovirulent strains of the Zika virus emerged with significant neurological involvement. Neurotropism refers to the ability of a virus to penetrate and infect the central or peripheral nervous system. Central nervous system (CNS) involvement can present with encephalitis/encephalopathy, impaired consciousness, myelitis, and posterior reversible encephalopathy syndrome, among others^4–6^. Secondary involvement of the CNS, including coagulopathic events such as ischemic or hemorrhagic events is also of concern. Peripheral nervous system involvement may include neuropathy, flaccid paralysis, and radiculopathy. Secondary demyelinating events, such as acute inflammatory demyelinating polyneuropathy, are also possible. Traditionally, cerebrospinal fluid (CSF) analysis in viral infections reveal lymphocytic pleocytosis, elevated albumin, and in some cases direct RNA measurement^7^.

Ongoing zoonosis of neurovirulent viruses are a threat and concern to public health systems throughout the world^8^. Furthermore, the identification of effective treatment strategies can help prevent disease progression and help in morbidity and mortality outcomes. This article aims to provide a comprehensive review on mechanisms by which viruses exert neurotropism. Furthermore, we also provide a broad mechanistic overview by which viruses present with neurological manifestations but evade direct detection in CSF.

## Methods

A comprehensive literature search was conducted using PubMed/PubMedCentral/MEDLINE. A gray literature search was conducted using Google Scholar, and an evaluation of the first 100 results was conducted. A combination of relevant keywords and Boolean operators were utilized including: (‘neurotropic viruses’ OR ‘neurotropic virus’ OR ‘neuroinvasive viruses’ OR ‘neuroinvasive virus’) AND (‘nondetection’ OR ‘undetected’ OR ‘undetectable’ OR ‘false-negative’ OR ‘diagnostic failure’) AND (‘cerebrospinal fluid’ OR CSF OR ‘spinal fluid’) AND (mechanism* OR ‘pathophysiological mechanism’ OR ‘pathophysiology’ OR ‘molecular mechanism’ OR ‘viral escape’). Results from nonpeer reviewed sources, nonEnglish records, and abstracts/conference posters were excluded. Both human and nonhuman studies were included. Records were exported to Microsoft EndNote X9 (bld 13 966), and duplicate records were excluded. Titles and abstracts were manually screened for exclusion and removed if determined to be irrelevant. This review was completed using the Scale for the Assessment of Narrative Review Articles (SANRA) guidelines.

## Mechanisms of neuroinvasion

**Figure 1:**
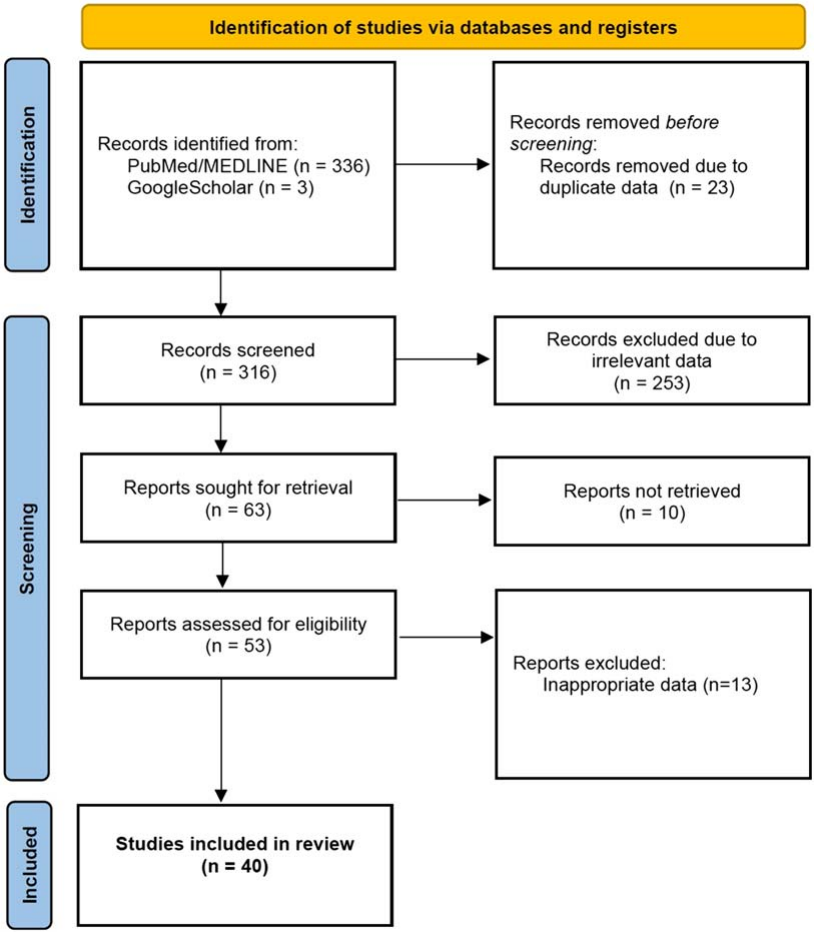
Flow diagram for search strategy.

Many of the records captured from this review specifically discussed arboviruses (e.g. *Flaviviridae, Togaviridae)* due to both the emergence and re-emergence of these viruses and the neurovirulence observed in newer strains. A flow diagram of the search results is included in Figure 1.

### Blood brain barrier dysfunction

The blood brain barrier (BBB) is a selectively permeable membrane that is formed between the endothelial cells of the brain capillaries. Endothelial cells are connected through tight junctions. Tight junctions consist of various transmembrane subunits including occludins, claudins, and junctional adhesion molecules^9^. The blood cerebrospinal fluid barrier (BCSFB) is in the choroid plexus of the brain ventricles. Similarly, to the BBB numerous tight junctions and adherent junctions regulate influx in the BCSFB. Fenestrations within the BCSFB allow for CSF production^10^.

Pathogen entry into the CNS through paracellular or transcellular means occurs through either direct inflammation and disruption of the BBB or through secondary cell-signaling events leading to dysfunctional tight junctions. The toll-like receptor-3 (TLR-3) also known as cluster of differentiation 283 (CD 283) is a protein encoded by the TLR-3 gene and plays a key role in pathogen recognition and activation of the innate immune system^11^. TLR-3 recognizes ds-RNA carried by some viruses. Recognition of RNA leads to activation of downstream cell-signaling processes including the nuclear factor kappa-light-chain-enhancer of activated B cells (NF-κB), and interferon regulatory factor 3 resulting in the production of inflammatory cytokines. Some neurotropic viruses may be present in the CNS at low concentrations, below the detection threshold of standard PCR-based methods. This low viral load can result in false-negative results during direct RNA detection in the CSF^12^.

TLR-3 has been implicated in the invasion of the CNS by the West Nile virus. In-vivo Tlr-3^-/-^ knock-out mice had increased peripheral WNV infection. However, viral infection and inflammation of the brain were markedly lower compared to the control. It is hypothesized that there is reduced immune-mediated neuroinflammation exerting a neuroprotective effect. TLR-3 deficiency leads to lower production of tumor necrosis factor-α and interleukin-6 in microglia. Increased neuroinflammation via TLR-3 may lead to BBB dysfunction and serve as an entry point for viruses into the CNS^13^. Similarly, chikungunya virus mouse models with a partially (IFN-α/βR^+/−^) or totally (IFN-α/βR^−/−^) abrogated type-I interferon signaling pathway was associated with severe infection including neuroinvasion. Chikungunya virus exerts neurotropism through disruption of the BCSFB and invasion through the choroid plexus^14^. Similarly, other factors associated with BBB dysfunction in arbovirus neuroinvasion include matrix metalloproteases, tyrosine kinase disruption, and other chemokine receptors. These specific examples highlight that neuroinflammation may contribute to or protect against BBB disruption and neuroinvasive viral disease^15^.

### Transneural invasion

The clinical manifestations of neuroinvasive viral disease include flaccid myelitis. The WNV, for example, has been implicated in anterior horn cell injury and subsequent muscle weakness. Retrograde axonal transport refers to movement from the axon to the cell body and is largely mediated by the protein dynein^16^. Numerous viruses have been implicated in the exertion of tropism through this mechanism, including rabies virus, poliomyelitis, and herpes simplex virus (HSV), among others^17^. Among arboviruses, Samuel *et al.* inoculated the sciatic nerve of hamsters with WNV and determined that neuronal invasion occurred through axonal transport. An axotomy successfully ablated further neuronal invasion and disease. Bidirectional axonal transport was also observed in this study^18^. Similarly, other arboviruses (Japanese encephalitis virus (JEV), tick-borne encephalitis (TBE) virus) have shown preference for motor nerve invasion and subsequent axonal transport to anterior horn cells^19,20^. The lack of sensory nerve involvement is likely due to virus-protein interactions and axonal kinetics. However, more studies are needed to substantiate this hypothesis.

Transneural invasion has also been implicated in the gut-brain axis. An outbreak of TBE was observed in Hungary due to the consumption of raw milk. Similarly, the pathogen is believed to infect intestinal epithelial cells and lymph nodes before eventual spread into adjacent neurons and subsequently the CNS^21^. The olfactory neurons and bulb have long been postulated as a mechanism of virus entry into the CNS due to their direct projection into the brain via the cribriform plate. Also, it facilitates virus transmission via airborne particles and respiratory droplets. Brown *et al.* ^22^ reported significant inoculation of the olfactory bulb of the WNV compared to other regions, including the brain in an in-vivo mouse model. Similarly, Ricklin *et al.* observed significant oronasal inoculation of the JEV and spread through oronasal secretion in pigs. Inoculation with JEV occurred in pig-to-pig transmission without an arthropod vector. Subsequent involvement of the olfactory bulb with inflammatory cells was observed^23^.

### Trojan horse mechanism

The ‘trojan horse’ mechanism is described as the viral invasion of an immune cell with subsequent crossing of the BBB leading to disseminated CNS invasion. Neuroinflammation allows increased BBB permeability and leukocyte cross into the CNS^24^. Indeed, knock-out models for certain chemokines and adhesion molecules, including ICAM-1, and CLEC5a have been shown to reduce peripherally trafficked immune cells within the CNS^25^. Furthermore, the release of chemokines allows for chemotaxis, thus promoting the CNS entry of immune cells. Previous studies have determined the susceptibility of immune cells to arbovirus invasion with trafficking across the BBB. Despite this, there are mixed results on the exact pathophysiologic mechanism and evidence of peripherally trafficked inoculated immune cells within the CNS. An in-vivo study in cattle determined that RNA viruses can enter the CNS via infected microglial precursor cells through a ‘trojan horse’ mechanism^26^.

**Figure 2:**
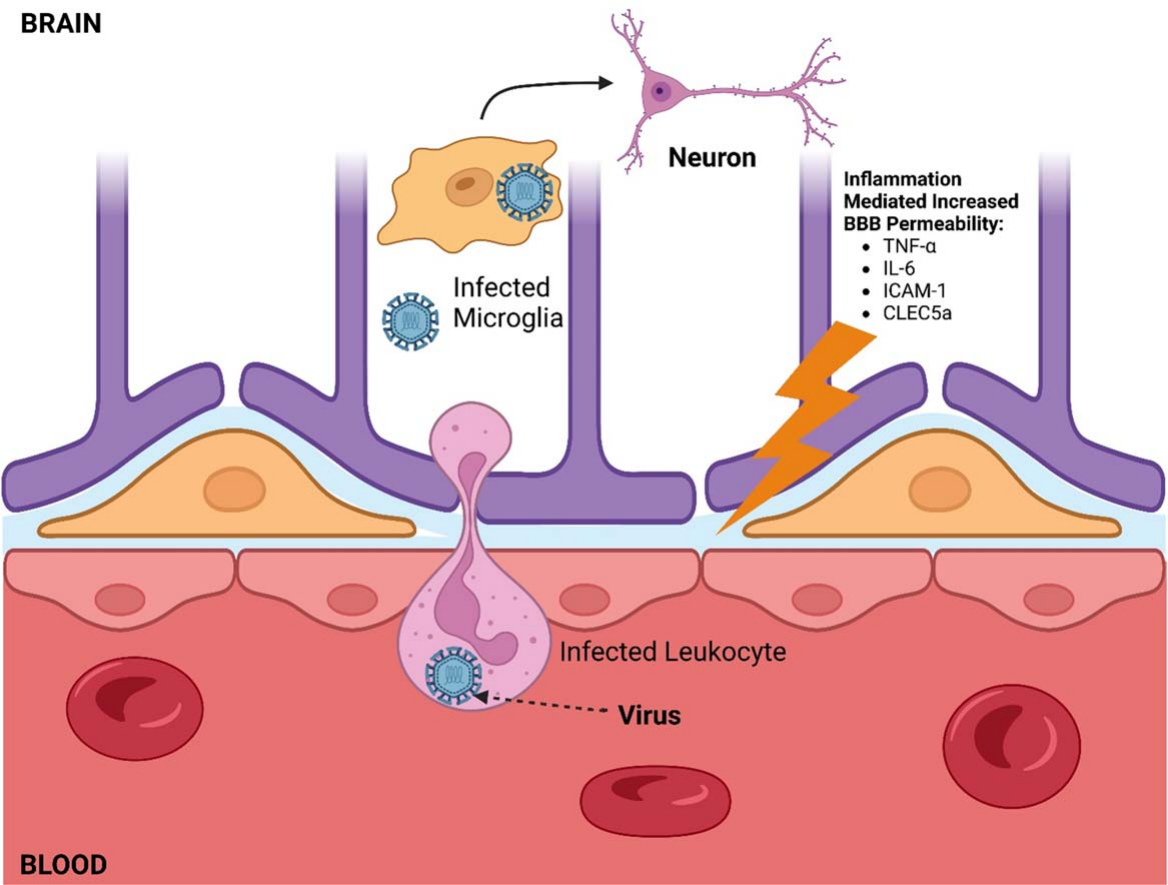
Virus infected leukocyte trafficking across the blood brain barrier (‘trojan horse’ mechanism) into the central nervous system.

Kumar *et al.* conducted an in-vivo study that examined diabetic mice infected with WNV and the subsequent infiltration of leukocytes into the CNS. They determined that CD45+ leukocytes and CD8+ T cells were significantly reduced in diabetic mice with an associated decrease in ICAM-1 and E selection expression. The decreased ability to clear the WNV infection was also observed in diabetic mice likely due to decreased penetration of immune cells into the CNS^27^. Therefore, immune cell prevalence in the brain is multifaceted and further research is needed in determining the neuroprotective and neuro-degenerative effects of immune cells in neuroinvasive viral infections. Difficulties arise in determining the origin of immune cells found in the CNS. Studies that specifically examine peripherally trafficked immune cells are needed. Figure 2 provides a visual representation of the ‘trojan horse’ mechanism.

## Mechanisms of CSF nondetection

### Intrathecal antibody production

Definitive hematopoiesis begins with B cell production in the primary lymphoid tissues of the bone marrow, including those in the skull cap^28^. These naïve immature B cells then migrate to secondary lymphatic organs such as the spleen to develop further as naïve mature B cells. B cells must pass through multiple checkpoints to evaluate for faulty self-reactivity. Reactive B cells will undergo ‘corrective processes’, which include clonal anergy, clonal deletion, and receptor editing^29^. Clonal anergy is a process to inactivate self-reactive B cell receptors; clonal deletion is the induction of apoptosis in self-reactive B cells; while receptor editing involves the rearrangement of light chain receptors to avert self-reactivity^30^. These proposed processes are the body’s natural defense mechanism to maintain immunologic self-tolerance.

The successful migration to secondary lymphoid tissues is then followed by sensitization: a process in which naive B cells are activated into antigen-activated B cells. Some of these B cells may then develop further outside of germinal centers as plasmablasts or within germinal centers as GC B cells^31^. GC B cells undergo somatic hypermutation and affinity maturation, a process that involves pruning and mutation to create B cells with strong affinities to their specific antigens^32^. In the event of infection, memory B cells derived from centrocytes and plasmablasts can rapidly differentiate into antibody-secreting plasma cells^29^. Various cytokine and chemokine secretions promote translocation and the production of immunoglobulins, inducing an immunological response.

Normally, B cells exhibiting the CD23 + phenotype can enter all parts of the human brain parenchyma in small amounts^33^. However, the breakdown of the BBB due to inflammation allows increased infiltration of B cells; thus, aiding the defense response within the CNS. The brain residing B cells and the infiltrating B cells will then synergistically synthesize virus-specific IgM, IgG, and IgG antibodies^34^. The results of experiments carried out in mice had shown that IgM-secreting B cells play a critical role in defending the CNS. Diamond *et al.* reported that eliminating IgM secretion leads to disseminated WNV infection with a 100% mortality rate in mice^35^. Therefore, early migration of IgM-producing plasma cells is critical to preventing mortality. This study was then replicated in other scenarios involving other flaviviruses; where B cells from JEV-infected, dengue-infected, and zika-infected mice were shown to provide protection against WNV^36–38^. Similarly, Kaiser *et al.* recorded intrathecal synthesis of immunoglobulins (IgM >IgG, IgA). They also observed higher CSF cell counts in those with severe TBE^39^.

The recruitment into the CNS after a ‘first hit’ allows these immune cells to naturally reside in the brain parenchyma to quickly produce antibodies in a possible ‘second hit’^34^. The implications of immune cell colonization in the CNS and rapid antibody production likely leads to improved immune-mediated responses in re-infection. Rapid antibody production and antigen neutralization may lead to difficulties in directly capturing viral RNA in CSF studies. In some cases, the host immune system may effectively clear a neurotropic viral infection from the CNS before diagnostic testing occurs. In these situations, the viral RNA may be undetectable in the CSF, even though the virus was previously present. The appearance of intrathecal antibody production against neurotropic viruses has raised concerns about the prevalence of primary CNS autoimmunity. It has been hypothesized that conditions like autoimmune encephalitis, multiple sclerosis, neuromyelitis optica spectrum disorder, and myelin oligodendrocyte-associated disorders may be triggered by the production of intrathecal antibodies against neurotropic viruses (such as Epstein-Barr virus)^40^. The lack of viral RNA detection in CSF may indicate low intrathecal viremia but does not exclude CNS invasion altogether^41^.

### Transneuronal propagation

Transneuronal virus transport occurs through multiple mechanisms: trans-synaptic transport and synaptic microfusion. Both mechanisms allow for virus propagation from a neuron-to-neuron fashion without direct penetration into the CSF. Thus, we use the term ‘neuroevasion’ to describe significant neurological signs and symptoms without evidence of direct CNS invasion. This is of particular concern and relevance given the ongoing coronavirus disease 2019 pandemic. As there have been multiple reports of CNS disease from severe acute respiratory syndrome coronavirus-2 without direct CSF evidence of the virus^42^. The first recorded transneuronal propagation was seen in the HSV. The HSV isolate was introduced into the rabbit cornea and subsequent encephalitis was recorded. Such viral transmission is described in both the rabies virus and the pseudo-rabies virus^43^. This viral mode of transmission implicates invasion into the CNS from peripheral nerves but can also be seen in neuron-to-neuron transmission in the CNS. Thus, intracellular viral spread occurs without the presence of the pathogen in the extracellular space^44^. This form of transneuronal spread has also been seen in arboviruses such as the Sindbis virus^45^. Studies have shown that the propagation of neurotropic viruses occurs through the virus using host anatomical transport pathways, including proteins such as dynein and kinesin. These neurotropic viruses are then postulated to enter synaptic vesicles with subsequent neuroinvasion of the postsynaptic neuron. Extracellular vesicle transport is also implicated in the neuro-degenerative processes of arboviruses such as Zika virus^46^. Transneuronal spread in the CNS also allows for evasion of intrathecal humoral immunity and subsequent antibody production. Despite this, intracellular viral antigens may still be detected using T-cell-mediated cytotoxic immunity. Further studies are needed to examine direct virus-host protein synaptic interactions to better understand the underlying molecular mechanisms and possible therapeutic targets.

**Figure 3:**
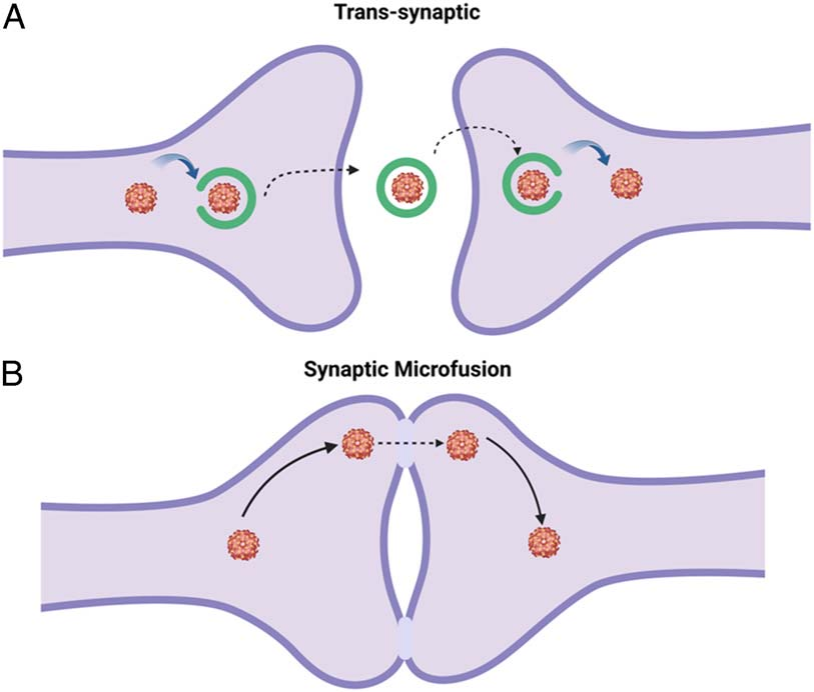
(A) Trans-synaptic virus transmission. (B) Synaptic microfusion and subsequent transneuronal virus propagation.

Synaptic microfusion refers to the physical fusion of the cell membrane of the synaptic terminal and postsynaptic dendrite serving as a conduit for transneuronal virus propagation. Modulation of the synaptic fusion inhibitory peptide and neurokinin-1 by the measles virus has been implicated in synaptic microfusion and subsequent transneuronal infection^43^. By manipulating these molecules, the virus can enhance synaptic microfusion and promote transneuronal infection. Similar to trans-synaptic spread, synaptic microfusion leads to intracellular virus propagation without entering the extracellular spread. Further studies on the molecular mechanism of virus-specific interactions within the axon and terminal synapse is needed to better understand how neurotropic viruses exert neurovirulence and evasion from CSF detection. Since the virus remains intracellular and does not enter the extracellular space, it is difficult to detect using conventional PCR techniques that rely on the presence of viral genetic material in the CSF. The absence of viral RNA or DNA in the CSF can lead to false-negative results and may hinder the accurate diagnosis and treatment of viral infections in the CNS. To overcome this diagnostic challenge, newer tests, such as cell-free DNA and RNA sequencing in CSF, can improve diagnostic yield^47,48^. Figure 3 provides a visual representation of both mechanisms of transneuronal propagation.

## Conclusions

The ongoing zoonosis of emerging neurotropic viruses (e.g. flaviviruses, togaviruses, and coronaviruses) are a continued threat to public health systems worldwide. Prior strains of flaviviruses (i.e. Zika virus) were not neurotropic. However, new neurovirulent strains have emerged with significant neurological manifestations. Neuroinvasion occurs primarily through transneural transmission, BBB dysfunction/breakdown, and infected immune cells (‘trojan horse’ mechanism). Neurotropic viruses often evade direct CSF detection through multiple mechanisms including transneuronal propagation, intrathecal antibody synthesis, and rapid neutralization. Consistent positive CSF viral cultures indicate enhanced neuroinvasion and may be associated with significant morbidity and mortality. However, the lack of CSF detection does not exclude CNS involvement due to the mentioned neuroevasive mechanisms. CSF findings in neuroinvasive disease often include signs of BBB dysfunction such as elevated protein, albumin, and IgG index. Lymphocytic pleocytosis, antibody detection, and positive viral RNA cultures may also be present. Future directions include focusing on investigating the entry mechanisms, host factors, and immune evasion strategies employed by viruses to infect the CNS while escaping direct RNA detection. Studies could examine viral replication in the CNS, specifically at the BBB, and the role of cellular receptors in facilitating viral entry. Additionally, research could explore the interplay between viral and host factors that may suppress viral RNA detection, such as viral proteins that interfere with host immune responses or the role of viral latency and reactivation in the CNS. Finally, efforts to develop more sensitive diagnostic methods for detecting low levels of viral RNA or alternative biomarkers indicative of viral infection could further our understanding of viral CNS infections and improve clinical management.

## Ethical approval

Not applicable.

## Consent

NA.

## Sources of funding

This manuscript was not funded.

## Author contribution

B.S.S.: intellectual conceptualization, writing-original draft preparation, writing-review and editing, and visualization; S.Z.: writing-original draft preparation, writing-review and editing; M.R.M., T.S., V.K., A.D., S.D., D.C.A., R.C.S. K.N., M.A.G.: writing-original draft preparation.

## Conflicts of interests disclosure

On behalf of all authors, the corresponding author states that there is no conflict of interest.

## Guarantor

Bahadar Srichawla.

## Availability of data and materials

Not applicable.

## Provenance and peer review

Not commissioned, externally peer reviewed.
